# Estimating the Diversity, Completeness, and Cross-Reactivity of the T Cell Repertoire

**DOI:** 10.3389/fimmu.2013.00485

**Published:** 2013-12-26

**Authors:** Veronika I. Zarnitsyna, Brian D. Evavold, Louis N. Schoettle, Joseph N. Blattman, Rustom Antia

**Affiliations:** ^1^Department of Biology, Emory University, Atlanta, GA, USA; ^2^Department of Microbiology and Immunology, Emory University, Atlanta, GA, USA; ^3^Center for Infectious Diseases and Vaccinology, School of Life Sciences, Arizona State University, Tempe, AZ, USA

**Keywords:** *αβ* T cell, repertoire, precursor frequency, cross-reactivity, pathogen recognition

## Abstract

In order to recognize and combat a diverse array of pathogens the immune system has a large repertoire of T cells having unique T cell receptors (TCRs) with only a few clones specific for any given antigen. We discuss how the number of different possible TCRs encoded in the genome (the potential repertoire) and the number of different TCRs present in an individual (the realized repertoire) can be measured. One puzzle is that the potential repertoire greatly exceeds the realized diversity of naïve T cells within any individual. We show that the existing hypotheses fail to explain why the immune system has the potential to generate far more diversity than is used in an individual, and propose an alternative hypothesis of “evolutionary sloppiness.” Another immunological puzzle is why mice and humans have similar repertoires even though humans have over 1000-fold more T cells. We discuss how the idea of the “protecton,” the smallest unit of protection, might explain this discrepancy and estimate the size of “protecton” based on available precursor frequencies data. We then consider T cell cross-reactivity – the ability of a T cell clone to respond to more than one epitope. We extend existing calculations to estimate the extent of expected cross-reactivity between the responses to different pathogens. Our results are consistent with two observations: a low probability of observing cross-reactivity between the immune responses to two randomly chosen pathogens; and the ensemble of memory cells being sufficiently diverse to generate cross-reactive responses to new pathogens.

## 1 Introduction

The clonal selection theory of adaptive immunity requires that the immune system is able to produce a large and diverse repertoire of immune cells (clones), with each cell expressing a receptor with different antigenic specificity (1, 2). Following infection, the few clones that are specific for the antigens expressed by the pathogen proliferate and differentiate into effector cells which control the infection. Subsequently, the maintenance of an increased number of these pathogen-specific cells results in long-lasting immunological memory (3–5). Accurate quantification of changes in the numbers of antigen-specific cells during infection and vaccination, together with advances in molecular and cellular biology, has allowed us to make considerable progress toward understanding the dynamics of the generation of immune responses (3, 6, 7) and the requirements for pathogen control (8, 9). Furthermore, deep sequencing technology has provided a first quantitative snapshot of the diversity of immune cells (10, 11). These technological advances set the stage for understanding the relationship between the diversity of immune cells (the repertoire) and immune protection from an extensive array of pathogens to which we are exposed.

We begin by outlining our current understanding of T cell receptor diversity and discussing problems associated with the quantification of the T cell repertoire. Next, we explore how diverse the immune system needs to be by exploring the relationship between the diversity of the T cell repertoire and its ability to provide protection from pathogens. Finally we consider how the degree of specificity of T cells (often defined by measuring how cross-reactive they are) affects the relationship between the repertoire and host response to a given pathogen. We focus on *αβ* T cells and the term “T cell” refers to the CD8 subpopulation of T cells unless we explicitly specify a different subpopulation.

We have intentionally used simple models and calculations because, in the absence of detailed information on the terms and parameters, simpler models frequently generate more robust qualitative results than complex models (12, 13). The focus of the paper is to highlight the limitations arising from uncertainties in current estimates of parameters, and in particular to gain maximum insight from the one key parameter – the precursor frequency of T cells specific for different epitopes – that can be accurately measured. Throughout this paper we emphasize current puzzles and problems and, where possible, suggest new approaches to solving them.

## 2 Measuring the Diversity of the T Cell Repertoire

### 2.1 What is the potential repertoire?

T cells develop from progenitor cells in the thymus where the germline T cell receptor (TCR) *α* and *β* genes undergo somatic recombination of the V-J and V-D-J gene segments, respectively (14, 15). The antigenic specificity of each T cell is determined by the amino acid sequence of these rearranged TCR genes, and in particular by the hypervariable complementarity determining region 3 (CDR3) that mostly account for direct contacts with peptides presented on major histocompatibility complex (MHC) proteins, and is encoded by the junction of the V, (D), and J gene segments (16). The diversity of generated TCR genes is therefore due to: (1) selection of one from a number of possible V, D, and J gene segments, (2) semi-random cleavage of recombination hairpin intermediates, and (3) N-nucleotide addition and subtraction at the junction of V, D, and J genes (17, 18). Finally, the pairing of different *α* and *β* chains to generate a functional receptor results in additional diversity (19).

How many different T cell receptors can be generated? The first steps toward understanding the magnitude of the diversity of the T cell repertoire came from the pioneering studies that identified the molecular mechanisms involved in the recombination of V, (D), and J gene segments and N-region diversification described above for the generation of the *α* and *β* TCR chains (14, 15, 19). It was estimated that these processes together with pairing between different *α* and *β* chains could give rise to around 10^15^ possible *αβ* TCR (19). The question of the potential number of TCR sequences has recently been revisited and significantly larger estimates for the diversity of the TCR *β* chain were obtained (20, 21). Murugan et al. (21) used statistical analysis of non-productive TCR *β* chain to conclude that the CDR3 (variable) region of the TCR *β* chain alone has a potential diversity of ~10^14^ different sequences. They used empirical *β* chain data to show that N-nucleotide insertions at the V-D and D-J junctions are uncorrelated, their length distributions are nearly identical and their lengths could exceed six nucleotides which was assumed in previous estimates (19). We might expect that a similar analysis would result in upward revision for the potential diversity of the *α* chain (though the estimates of diversity would increase less than for the *β* chain because the *α* chain has only one V-J junction). This will result in a truly enormous potential repertoire of over 10^20^ for the *αβ* TCR.

### 2.2 What is the realized repertoire in an individual?

Only mature T cells that have passed thymic selection (naïve T cells) can be employed in immune responses for protection against pathogens. Thus, in order to understand the balance between diversity and protection, the most important measurement is the “realized” T cell diversity in an individual (i.e., the actual number of different TCR in the mature T cell compartment).

The diversity of the naïve T cell repertoire was initially estimated prior to the advent of deep sequencing technologies by the use of spectrotyping, which involved amplifying mRNA from particular V-J sequence combinations, separating the amplified products on the basis of size, and exhaustive conventional sequencing of a particular length CDR3 product. The diversity of TCR sequences in this sample was then extrapolated to the total T cell population.

#### 2.2.1 TCR diversity and clone size in humans

Arstila et al. (22) used spectrotyping to estimate that there are 10^6^ *β* chains in the blood each pairing on average with at least 25 different *α* chains, and consequently proposed a lower bound to the estimate of the T cell repertoire in humans of around 2.5 × 10^7^ specificities. Advances in deep sequencing have confirmed that estimation of *β* chains is in the range of 1 − 4 × 10^6^ (20, 23, 24).

There is however considerable uncertainty about the extent to which 2.5 × 10^7^ specificities underestimates the diversity of T cells in humans (25, 26). A repertoire of 2.5 × 10^7^ suggests a naïve clone size on average of over 4 × 10^3^ cells (>10^11^/(2.5 × 10^7^)). This could happen if each clone gets produced multiple times or if once produced a given clone would undergo about 12 rounds of division. The first scenario is unlikely, given the very large estimates of potential diversity (19–21). If the second scenario happens, it must occur in the periphery. Expansion of clones in the thymus would result in a much lower frequency of detectable T cell receptor excision circles (TRECs) in the naïve pool of recent thymic emigrants than is currently observed (27–29). Arstila et al. points out that naïve T cells in the periphery could divide more than 12 times during a human lifespan (26). However, as the total number of naïve T cells remains relatively stable (because division is balanced by death) changes in clone size would have to arise from stochastic drift and this seems unlikely.

#### 2.2.2 TCR diversity and clone size in mice

Interestingly, it was estimated that TCR *β* chain diversity in mouse spleen is quite similar to the one measured in human blood. The *β* chain repertoire of 5 − 8 × 10^5^ specificities with each variable domain of *β* chain sequence being shared by 30–40 T splenocytes have been reported using spectrotyping technology (30). Pairing with *α* chain was estimated to add a factor of 2.4 and resulted in total *αβ* TCR diversity of 2 × 10^6^. Taking into account that there are 2 × 10^7^ splenocytes it was estimated that the clone size of *αβ* TCR is equal to 10 cells (30). The bias in recombination and *α*-*β* TCR pairing will likely affect the T cell clone size. A recent study that enumerated the number of naïve T cells specific for different epitopes suggests that there are between 15 and 1500 unique cells in the mouse spleen specific for any given epitope, implying that the number of naïve cells with a given TCR *α*-*β* combination is very small, and indeed that most clonotypes have clone size of one (31, 32). This is in contrast with the earlier estimates that suggest an average clone size of 10 cells/clone in the spleen (30). Consequently, it brings the repertoire in the spleen toward the total number of naïve T cells in the spleen, and increases the lower bound of the total *αβ* T cell repertoire in the mouse by an order of magnitude. In this case the estimate of 2 × 10^7^ specificities becomes very close to a lower bound estimation for human T cell repertoire.

#### 2.2.3 Limitations in estimates of realized diversity

Current estimates of the realized diversity are lower bounds. The limitations of these studies is the lack of information on the pairing of different TCR *α* and *β* chains. Bulk sequencing of a single chain, or even of both TCR *α* and *β* chains, is not sufficient to inform us of the potential diversity (33). In principle this problem could be comprehensively addressed by single cell sequencing that would obtain linked *α* and *β* chain sequences, but this remains technically and financially infeasible for the large sample sizes required to evaluate naïve repertoires with high diversity (34); the cost of single cell sequencing remains at $1 per cell, making the analysis of T cells from a single mouse more than a $10 million experiment! Oil emulsion lysis strategies (35) combined with micro-sequencing have increased the capacity of such single-cell studies, but these still are only able to capture <1% of the total naïve T cell repertoire in a single mouse. New techniques or methods need to be developed.

**Figure 1:**
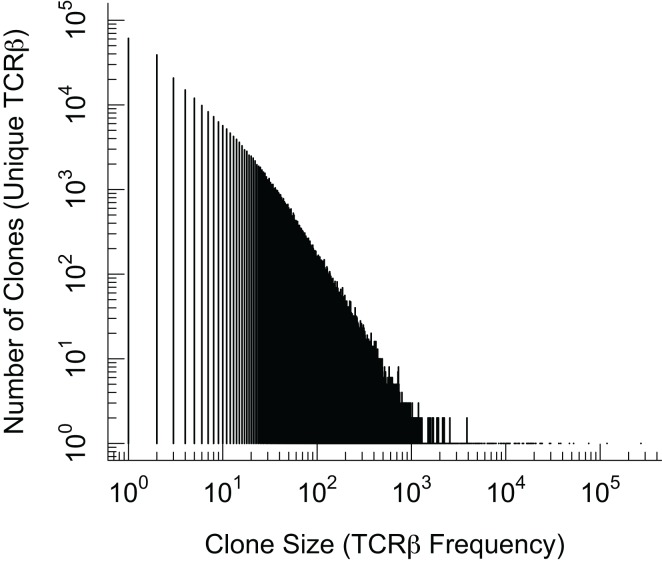
**Plot of the frequency distribution in the *β* chain sequences of naïve CD8 T cells**. Naïve (CD44*^lo^*) CD8 T cells from C57Bl/6 mice were isolated by magnetic beads and >98% purity confirmed by flow cytometry. Genomic DNA was subjected to TCR*β* V-J multiplex DNA sequencing and the distribution of unique in-frame CDR3 sequences is plotted. We note that the term “clone” on the x and y axis labels refers to clones based on *β* chain sequences alone.

In order to have an accurate and comprehensive quantitative description of diversity, it is important to define what we mean by diversity. We can describe T cell repertoire diversity in terms of summary measures of diversity borrowed from the ecological literature. This includes enumerating the number of distinct clones or computing the Simpson diversity index (36) that takes into account the number of clones and their frequencies. However these summary approaches compress all of the diversity information into a single number. A more comprehensive statistical approach retains the frequency distribution of different clone sizes. In Figure 1 we show a plot of the frequency distribution of *β* chain sequences in the mouse naïve T cells using preliminary data. We find a majority of *β* chain sequences are present at low frequencies and fewer sequences occurring at much higher frequencies. A key problem is that we do not know the *α* chain sequences pairing with each of these *β* chains, and this restricts our ability to infer diversity of T cells from these observations.

Several clones in Figure 1 have very high frequencies and the exact underling mechanisms are not known. The sequences which are more common (generated more frequently) are more likely to be shared between different individuals. It was reported that inbred mice and individuals with the same MHC share some T cells with identical receptors (11, 20, 37, 38). These constitute “public” T cell clones, in contrast with the majority of the T cell clones that are unique to an individual and comprise the “private” part of a repertoire response. In general, the frequency of public TCR clonotypes exceeds what is expected if T cells were chosen at random with equal probability from the total potential repertoire, and perhaps not surprisingly, the clone size of public T cells is higher than that of private T cells in the naïve T cell repertoire [(33); Blattman et al. unpublished results]. This has been suggested to arise due to MHC restriction during thymic selection, biased frequencies of recombination, as well as degeneracy in the genetic code which allows more than one nucleotide sequence to give rise to the same amino acid sequence (33, 39). The factors involved in the evolution and/or selection of public T cell clonotypes and their possible role in the control of infections remain puzzling questions.

In summary we have estimates of the potential repertoire of upward of 10^20^ TCR. The estimates of the realized repertoire suggest lower bounds of 2.5 × 10^7^ and 2 × 10^6^ in humans and mice. Two puzzles which we will address are: why humans and mice might have similar repertoire sizes (Section 3.2); and why the potential repertoire so greatly exceed the realized repertoire (Section 3.3).

## 3 Understanding Diversity, the Repertoire and Cross-Reactivity

In this section we use quantitative calculations to explore the consequences of the observations on the repertoire described in the previous section. We begin by looking at whether the diversity of the repertoire may be explained by the relationship between diversity and protection. We then address questions associated with our current understanding of repertoire diversity and cross-reactivity.

### 3.1 Relationship between diversity and protection

Clearly a large repertoire is required to generate a T cell response to a diverse array of pathogens. However, to our knowledge, few empirical studies consider the relationship between the repertoire and protection. To some extent the paucity of experiments on this topic is because of difficulties in quantifying the repertoire (see earlier discussion). Studies on mice, expressing a single fixed transgenic TCR chain (either *α* or *β*) that measure the number of different paired endogenously recombined TCR chains, have shown that pairing is not completely random, as these mice express repertoires of reduced diversity and altered V gene usage (40–43). However, even in these mice there is still sufficient diversity to generate effective, albeit reduced, responses to control pathogen infections.

A relatively simple calculation can be made to estimate how diverse the TCR repertoire needs to be in order to provide reliable protection following infection with a pathogen. To provide protection against a pathogen there must be some number of clones present in the repertoire that are specific for that pathogen. Here, we extend the logic outlined in (44, 45). Let *R* be the T cell repertoire and let *p_i_* be the probability that a randomly chosen TCR binds to *i^th^* of the *k* epitopes derived from a given pathogen (*i* = 1:*k*). Note that *p_i_* is also equal to the precursor frequency of T cells for *i^th^* epitope. A pathogen is not detected if all *R* naïve T cell clones fail to recognize it, and this will happen with probability.

(1) $$\begin{array}{llll} P_{E} & ={\left(1-p_{1}\right)}^{R}{\left(1-p_{2}\right)}^{R}...\;{\left(1-p_{k}\right)}^{R}\; & & \;\\ & \approx \exp\left(-p_{1}R\right)\exp\left(-p_{2}R\right)...\;\exp\left(-p_{k}R\right)=\exp\left(-R\overset{k}{\underset{i=1}{\sum }}\;p_{i}\right) \end{array}$$

which gives

(2) $$R=\frac{-\ln\left(P_{E}\right)}{\sum_{i=1}^{k}\;p_{i}}$$

**Figure 2:**
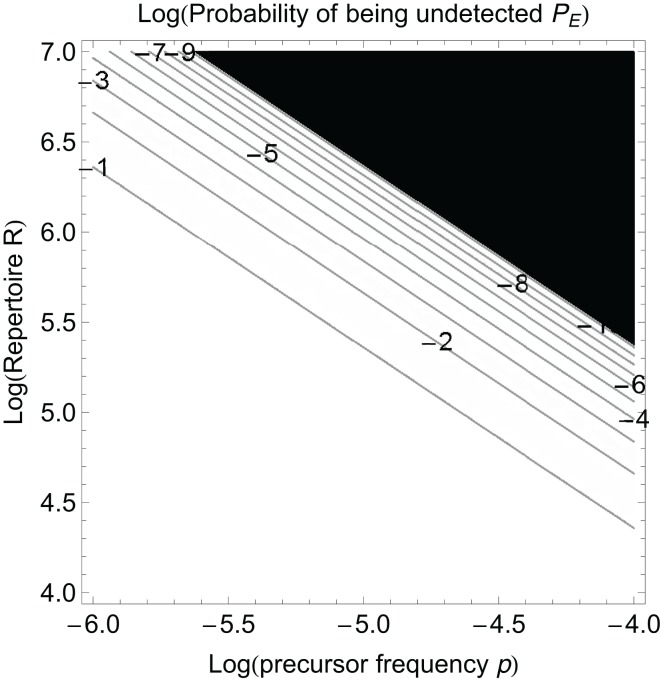
**The probability a pathogen is not detected, *P_E_*, as a function of the log of the precursor frequency *p* and the log of the naïve T cell repertoire *R***. The numbers on the contour lines in the plot indicate log *P_E_* values. Black color corresponds to the values of *P_E_* below the threshold of 10^−10^.

Equation (2) tells us how big the repertoire must be to detect at level *P_E_*. Figure 2 shows how the probability that a pathogen is not detected by the immune system depends on the repertoire size and the total precursor frequency *p* = ∑ *p*_*i*_. There is a very rapid decline in the probability of not being detected once the product of *p* and *R* becomes sufficiently large. We should note that *P_E_* is often termed as the “probability of escape” but it should not be confused with the usage of the term “escape” that refers to the generation of escape mutants in T cell epitopes after recognition has already occurred following infections such as HIV.

**Figure 3:**
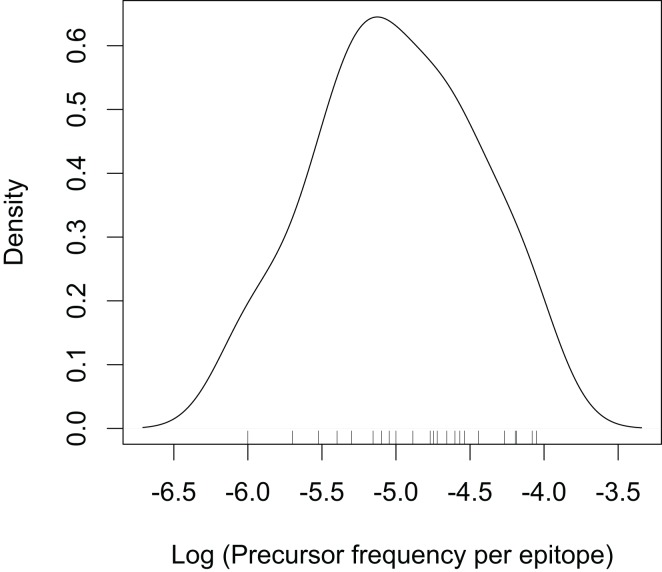
**Density distribution plotted from the precursor frequencies of naïve CD8 T cells for different epitopes reported in (31)**. The tick marks on the top of the x-axis indicate individual epitopes. Note the log scale on the x-axis.

**Figure 4:**
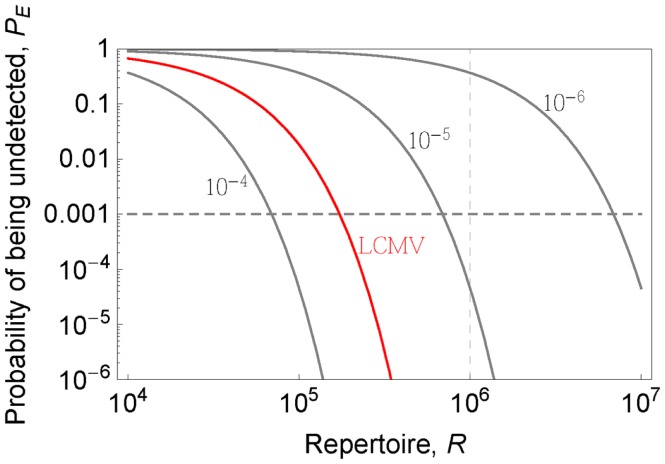
**Probability that a pathogen is not recognized *P_E_* (y-axis) is plotted as a function of the repertoire (x-axis) for indicated pathogen-specific precursor frequencies (gray lines)**. LCMV case (*p* = 4 × 10^−5^) is shown in red color.

If we know the precursor frequencies for pathogen epitopes and total number of epitopes we can relate the probability of being not detected to the repertoire *R*. We have much more accurate estimates for precursor frequencies against specific epitopes than for repertoire sizes (31, 46, 47). A recently developed method that combines pMHC tetramer staining with magnetic particle-based cell enrichment allows for the direct measurement of the frequency of naïve cells to different epitopes for both mice and humans (31, 48). Figure 3 shows the density distribution of naïve T cell precursor frequencies for different CD8 T cell epitopes in mice determined by this cell enrichment method using MHC tetramers complexed with different class I-restricted peptides (31). The total number of responded cells per mouse (naïve precursor frequency multiplied by total CD8 T cell number) varied from 15 in response to the L-338:*D^b^* epitope of LCMV to 1500 in response to an epitope from the murine cytomegalovirus (31). These estimates concur with previous *in vivo* estimates of precursor frequencies. These studies transferred different numbers of naive epitope-specific T cells and measured the proportion of the response arising from expansion of host versus donor cells following virus infection (46, 47). The effect of changing precursor frequencies on the probability of been undetected, *P_E_*, is given by equation (2) and plotted in Figures 2 and 4. Note, that precursor frequencies plotted in Figure 3 are likely biased toward immunodominant epitopes. Immunodominance is a complex issue, and the major factors that affect the magnitude of the T cell response to a particular epitope include: the processing and presentation of peptide on MHC (i.e., the amount of epitope); the number of precursor cells for this epitope; their affinities for the epitope; the extent of their recruitment and competition between the T cells for this and other pathogen epitopes (31, 49–51).

### 3.2 Scaling and the concept of a “protecton”

We now consider the scaling of the repertoire with the size of the organism. A few pathogen-specific precursors in a tadpole are likely to be able to mount a faster and more effective response than the same number of cells in an elephant (52). Langman and Cohen proposed the basic functional unit, the “protecton,” capable of providing robust protection. They suggested a tadpole (smallest vertebrate) has a single “protecton,” and the number of “protectons” scales with the size of an organism. Localized infections are surveyed by a draining lymph node rather than the entire immune system and thus we expect this unit should contain at least one “protecton.” Clearly the calculations for *P_E_* (how diverse the immune system needs to be to recognize pathogens) pertains to the “protecton” [see equations (1) and (2)].

Lets estimate the diversity in a “protecton.” Figure 4 shows how the probability of being undetected depends on the size of the repertoire *R* for different total precursor frequencies. The precursor frequency of T cells specific for a pathogen is, to a first approximation, the sum of the precursor frequencies for that pathogen’s different epitopes presented by MHC proteins. This can be estimated for LCMV by combining reported naïve precursor frequencies for few measured epitopes (31) and measurements of the fraction of the total LCMV specific responses which is directed against these epitopes (53). This gives us a total precursor frequency for LCMV specific T cells equal to ~4 × 10^−5^, and from Figure 4, a level of protection *P_E_* = 10^−3^ (i.e., 1 in 1000 “protectons” will fail to recognize LCMV) requires the repertoire in the “protecton” to be about 1.7 × 10^5^. In order to know the level of protection against diverse pathogens we need to know the distribution of precursor frequencies to pathogens. The existing data gives us lower bounds (because only cells specific for a few epitopes are measured) to the precursor frequencies of viruses such as MCMV (~1.3 × 10^−4^), Influenza (~4 × 10^−5^), Vaccinia (~1.1 × 10^−4^), RSV (~4.5 × 10^−5^), HSV (~2.9 × 10^−5^), and VSV (~10^−5^) (31). The precursor frequencies for those viruses are comparable or greater than that for LCMV with the exception of VSV and HSV for which only single epitope data were reported in (31). If this trend holds (i.e., the precursor frequency per pathogen is >10^−5^) it might suggest that having a repertoire of 7 × 10^−5^ is sufficient to give robust protection at the level *P_E_* = 10^−3^, and thus define the size of the “protecton.” For *P_E_* one order of magnitude lower and higher, i.e., *P_E_* = 10^−4^ − 10^−2^ will require a repertoire of ~9 × 10^5^ − 5 × 10^5^, suggesting that our estimate is quite robust to changes in *P_E_* (see Figure 4). We can expect the area of local surveillance (a small lymph node) in mice to have at least this number of different T cells.

How much bigger should the total repertoire size be so that the area corresponding to one “protecton,” randomly filled with T cells from the total circulating cells, has a relatively low number of clone repeats? We estimated that if *f* is a fraction of clone repeats in the “protecton” area with *m* cells, the total repertoire size *R* is bounded as (1 − *f*)(*m* − 1)/(2*f*) < *R* < (*m* − 1)/(2*f*). For example, for 5 or 10% of clone repeats in *m* we will have a multiplication factor for *m* for the total diversity in the ranges ~9.5 − 10 or ~4.5 − 5, respectively. To derive this formula we used two assumptions: first, the clones are equal in size and second, the size of total repertoire multiplied by clone size is much bigger than the size *m*. These calculations show that the total diversity doesn’t need to be much higher than the diversity in a “protecton.”

Several theoretical papers previously estimated that the repertoire of B and T cells scales as *ln*(*cM*), where *c* is a constant and *M* is the mass of an organism (45, 54). It was also estimated that humans should have B cell repertoire 2–5 times larger than mice (45) and similar reasoning could be apply to T cell repertoire. The diversity of the repertoire is linked to clone size and it was estimated that the size of T cell clones should scale as *M* and, correspondingly, the total number of T cells should scale as *Mln*(*cM*) (45). Wiegel and Perelson’s estimate shows that the repertoire in a human need not be much higher than that in a mouse even though the number of naïve cells in these organisms differs by over 10^3^ fold [mice have ~10^8^ T cells (30, 46) and humans between 10^11^ and 10^12^ T cells (22, 55, 56)].

Another reason for why humans need a more diverse repertoire than mice pertains to the number of pathogens to which they are exposed. As humans live longer than mice, other factors being equal, they will be exposed to more pathogens and require a lower *P_E_*.

### 3.3 Evolutionary considerations: Why encode such a diverse potential repertoire?

The calculations described in the previous section are consistent with the diversity of the repertoire that is observed in mice and humans (22, 30) (lower bound diversity in the range of 2 × 10^6^ to 2.5 × 10^7^ unique *αβ* T cells), and the diversity is sufficient to generate a low probability that a given pathogen is not detected (*P_E_* < 10^−4^). What those estimations don’t explain is why the immune system is able to generate a potential diversity of more than 10^15^ T cell specificities (19–21) that is vastly in excess of the realized repertoire?

Let’s consider a number of potential explanations for why the potential repertoire needs to be much larger than the realized repertoire. One simplistic explanation takes into account the observation that most of the generated progenitor T cells are deleted during positive and negative selection in the thymus. If a fraction *f* of the T cells generated in the thymus gets selected (i.e., pass positive or negative selection) then the potential repertoire should be (1/*f*) times the peripheral repertoire. Since only 3–5% of T cells pass thymic selection (57, 58), the potential repertoire need only be at most 33-fold higher than the realized repertoire, thus ruling out this explanation.

A second potential reason is the need to successfully recognize peptides in the context of the hundreds of MHC alleles in the population. The reported extent of thymic selection (see previous paragraph) allows us to reject this hypothesis – different cells may be selected in different MHC backgrounds but in all cases 3–5% of T cells pass thymic selection.

A third potential reason is the need to prevent pathogen escape mutations – mutations in an epitope that prevent it from being recognized by the immune system. To a first approximation having more than one epitope is the key factor that prevents escape – if the pathogen has *k* epitopes the probability of escaping all epitopes declines as *μ^k^*, where *μ* is the probability of mutation leading to the loss of one epitope. The number of epitopes to which a response is generated involves many factors such as immunodominance, MHC diversity, and T cell diversity. The relationship between these quantities is not understood and we do not know the contribution of T cell diversity to immunodominance due to problems in estimating TCR diversity described above. However from Figure 4 we note that the repertoire is sufficiently large to enable robust detection of subdominant epitopes in a biologically reasonable range of precursor frequencies [Figure 2; (31)].

A fourth potential reason considers the temporal aspect and changes in the repertoire over the lifespan of an individual. Thymic emigration results in a constantly changing repertoire over time. The total number of different T cells present in the individual over its lifespan could be much greater than its repertoire at any given time. In humans, for example, if we assume that thymic emigration is of the order of 10^7^ − 10^8^ cells per day (59, 60) then the realized repertoire over a lifespan might be as much as 10^12^ specificities which is much closer to the potential repertoire. There are two problems with this approach. First, it does not explain why mice have about the same potential repertoire as humans since a similar calculation for mice would result in a realized repertoire over the lifespan several orders of magnitude lower than humans. This is because both mice thymic output of the order of 10^6^ cells per day (61–63) and lifespan are smaller than for humans. Second, protection is related to the repertoire at a given time point. Changing the particular clones in the repertoire over time does not help unless the relevant clones are present at the time of infection or generated during the infection and consequently able to help with clearance of the pathogen. The continual influx of cells of new specificities is thus unlikely to be of significance for acute infections which are relatively brief, but has been suggested to contribute to the maintenance of the response during persistent infections (64–66). In the case of persistent infections, however, an occasional new pathogen-specific clone is unlikely to clear the infection if the much larger number of clones at the onset were not able to do so – and the new clone is likely to be exhausted rapidly. Finally, temporal aspects could change the total repertoire if we consider the sum of both naïve and memory compartments. As naïve cells convert to memory cells each time we confront an infection, the replenishment of naïve compartment with the cells of new specificities would increase the total repertoire (naïve plus memory compartments). However, even if the memory compartment is as diverse as the naïve, the total diversity would increase at most by a factor of 2 in comparison to the naïve compartment alone. Taken together we don’t expect temporal aspects to account for the differences in the sizes of the potential and realized repertoires.

We now describe a new evolutionary explanation that we call “evolutionary sloppiness.” The process of generation of diversity by recombination and *N* nucleotide addition and deletion are sloppy processes. To reduce the amount of diversity that can be generated might require putting additional costly constraints on these processes. This would explain why organisms are able to generate far more TCR diversity (in excess of 10^15^ TCR) then is needed. Finally we note that not all aspects of biology result in perfectly optimized solutions (67).

Additionally, it has been suggested that the thymus is an energy- and resource-expensive organ (68) but these energetic costs have yet to be quantified. Energetic costs to cell production and thymic selection would favor expansion of clones after thymic selection (i.e., to have an amplifier). This amplification could take place in the thymus or periphery and would scale with the size of the organism. This would result in clone sizes in men being ~1000-fold higher than in mice, which is unlikely (see discussion on TRECs in Section 2.2.1). Accurate estimates for clone sizes in humans and mice should allow us to resolve this question.

### 3.4 T cell cross-reactivity

Cross-reactivity is related to the observation that a given T cell can respond to more than one epitope, including epitopes that show strong sequence homology or completely unrelated (69–76). As might be expected the frequency of the former is higher than that of the latter. Flexible TCR-pMHC binding sites were suggested as a possible structural explanation for known high degree of *αβ* TCRs cross-reactivity to different pMHCs (77–80). Cross-reactivity can also arise in T cell clones with incomplete allelic exclusion at the *α* chain loci resulting in one *β* chain pairing with two different *α* chains. An upper bound on the frequency of such clones was estimated to be 30% (81–83).

The pioneering experiments of Selin and Welsh (69) found that the CD8 T cell responses of mice to pathogens such as the Pichinde virus (PV), Vaccinia virus (VV), and Lymphocytic choriomeningitis virus (LCMV) showed high levels of cross-reactivity. They found that prior vaccination with one of these viruses expanded a specific CD8 T cell subset that could be boosted during stimulation by the other viruses and showed an unexpectedly complex relationship between the responses to different viruses with asymmetry depending on the order of viral exposure (infection A followed by B stimulated different cross-reactivity than B followed by A) (69, 71, 84). In a very recent paper (80) the cross-reactivity studies were extended to analysis of the CD4 T cell repertoire against pathogens to which individuals had never been exposed. Surprisingly, they found that a large fraction of the CD4 T cells specific for these pathogens exhibited a memory phenotype and suggested that they had been generated by cross-reactive responses to other previously encountered pathogens including heterologous infections or environmental antigens.

The extent of cross-reactivity between the immune responses to different pathogens is of practical importance. Murine studies have not only demonstrated the presence of T cells that could cross-react between different pathogens such as PV, VV, and LCMV, but also showed that this cross-reactivity affected pathogenesis during subsequent infection (84–86). If these occurrences of cross-reactive responses are rare, then the examples above are simply interesting curiosities. If, on the other hand, cross-reactivity is common then we would need to move from our current paradigm, which looks at each infection independent of other infections, to a more complex view that incorporates the terms for the interactions between the immune responses to different pathogens. Thus a key step is to quantify the extent of cross-reactivity in the immune responses to different pathogens.

How can we predict the level of cross-reactivity between two pathogens? The current approach is based on the observation that number of possible peptide-MHC complexes is much larger than the total number of T cells, suggesting that a given T cell must be able to recognize many different peptide-MHC (i.e. have a high level of cross-reactivity) (87, 88). However it is not clear how these parameters can be measured. We propose an alternative calculation that allows estimation of the extent of cross-reactivity from the precursor frequency of T cells for pathogens – a parameter that can be reliably determined. Using slightly modified notation from (87) we first define four parameters:

*R* Repertoire (the number of clonotypically different naïve T cells in the repertoire)
*r* The number of different T cell clonotypes that will respond to the same peptide
*N* The total number of potentially immunogenic foreign peptides in the environment
*n* The number of different peptides to which a single T cell clonotype will respond

These four parameters are linked by the conservation equation (87):

(3) rN=nR

Lets suggest that a given pathogen has *k* epitopes to which T cells can mount a response. For a given T cell, the probability to recognize at least one epitope from a given pathogen could be written as:

(4) $$1-{\left[1-\frac{n}{N}\right]}^{k}$$

The probability that the same T cell will recognize at least one epitope on each of two pathogens with *k* epitopes (i.e., will be cross-reactive) will be a square of expression equation (4) and the probability to find at least one cross-reactive clone is equal to:

(5) $${\left[1-{\left[1-\frac{n}{N}\right]}^{k}\right]}^{2}\times R$$

Note, that in derived equation (5) we don’t know the parameters *k*, *n*, and *N* which makes it very difficult to apply directly. Interestingly, for one epitope (*k* = 1) and with application of cross-reactivity equation (3) the equation (5) simplifies to:

(6) $${\left[1-\left[1-\frac{n}{N}\right]\right]}^{2}\times R={\left[1-\left[1-\frac{r}{R}\right]\right]}^{2}\times R={\left[\frac{r}{R}\right]}^{2}\times R$$

Under the assumption that all naïve T cells able to respond to a given epitope are clonotypically different, which is supported by recent data (31, 80), we can think of *r*/*R* as a precursor frequency for a given epitope. The problem of estimating cross-reactivity in this case will be similar to the problem of estimating the probability of randomly choosing a two-colored ball from an urn when the frequencies of each of two colors are known. Interestingly, the measured naïve precursor frequencies for different immunogenic epitopes are similar for mice and humans and range from 1 to 100 cells per million cells (31). According to equation (3), this similarity immediately implies that cross-reactivity of each T cell receptor, *n*, is in the same range for mice and humans.

For the case when k > 1, we could not directly use the formula (5), due to unknown parameters, but can use a simple probabilistic calculation based on sampling multiple colored balls. We can write the frequency of cross-reactive cells between two randomly chosen pathogens (A and B) in terms of the precursor frequencies of T cells to these two pathogens, *p_A_* and *p_B_*, and the total number of cells T:

(7) $$\text{Expected number of cross-reactive cells}=p_{A}p_{B}\times T$$

and if we have clones of same size equal to *T*/*R*,

(8) $$\text{Expected number of cross-reactive clones}=p_{A}p_{B}\times R$$

where *R* equals the repertoire (the number of different T cell clones in each individual). When the average number of cross-reactive clones is less than one, equation (8) gives us the probability of observing a cross-reactive response between two pathogens in a single individual. We can use this framework together with the data on precursor frequencies (31) described in Figure 2 to get an estimate of the extent of cross-reactivity between the responses to unrelated pathogens. As described earlier we have an approximate precursor frequency per epitope of 10^−5^, and a precursor frequency per pathogen, with LCMV as an example, of about 4 × 10^−5^. If we assume there are about 2 × 10^7^ naïve CD8 T cells per mouse (89) then the number of cross-reactive cells between two unrelated pathogens will be ~0.032, which suggests cross-reactivity is very rare (a single cross-reactive clone will be found <4% of the time). In order to observe cross-reactivity between two random pathogens in a mouse we would need to have a precursor frequency per pathogen of at least 2.2 × 10^−4^, assuming that precursor frequencies are similar for both pathogens. There are quite a few reported examples of cross-reactive T cell responses to different pathogens. In addition to the experiments of Selin and Welsh (69), cross-reactivity has been reported between influenza virus and hepatitis C virus (72), EBV (73) or HIV (74), LCMV and vaccinia virus (75), and coronavirus and human papillomavirus (76). It remains to be seen if the observed cases of cross-reactivity arise from a reporting bias (failure to observe cross-reactivity between two pathogens is unlikely to be reported) or because some of the assumptions of our model are incorrect and need to be modified. For example, we assume all T cell clones have the same level of cross-reactivity – and introducing heterogeneity may dramatically increase the chances to observe cross-reactivity.

Even if cross-reactive T cell responses to two specific pathogens are rare, the accumulation of many successive infections could result in fairly frequent cross-reactivity between a new pathogen and sum of all the pathogens the individual has previously encountered. This is what was observed when T cell precursor frequencies were measured for novel pathogens in blood from adults (80). The precursor frequency for cells recognizing a self-antigen or an unexposed viral epitope was the same as earlier estimated in mice and humans (31) and ranged from one to ten cells per million of CD4 T cells. The surprise was that over half of the precursor cells specific for novel pathogens such as HIV (to which an individual had never been exposed) were of the memory phenotype (80), suggesting that they may have arisen as a consequence of exposure to a different previously encountered pathogen(s). Alternatively, these memory cells could be pseudo-memory cells acquired via the process known as “homeostatic proliferation” and driven by interaction with low-affinity self pMHCs that previously induced positive selection (90–94).

The Su et al. paper (80) raised an interesting question: why do memory cells invariably contribute about 50–80% of the precursors to pathogen the individual has never encountered? One possibility is that the memory repertoire is sufficiently large to be “complete.” In this case if we draw the same amount of cells from either naïve or memory compartments (or mixture from both) we will have the same precursor frequency for a pathogen. Then the relative contribution of naïve and memory cells to precursors is equal to their relative frequencies, and is scaled by the stimulation threshold which is known to be lower for memory cells.

We note that our equation (7) allows an estimation of cross-reactivity for unrelated pathogens or peptides and, based on reported precursor frequencies for different epitopes, we expect cross-reactivity to be rare. Several studies allowed to estimate the rate of cross-reactivity for closely related peptides. Su et al. (80) identified potential pathogens responsible for generating T cells cross-reactive to HIV in HIV-negative individuals as follows: they generated clones from the HIV precursors and identified two epitopes to which these clones were specific. Then using a BLAST search of pathogen sequences they identified 24 sequences similar to the two HIV epitopes. About 21% of the HIV clones responded to two of the BLAST sequences corresponding to environmental pathogens. This number is comparable to result obtained in the earlier study, which showed that although the majority of 171 generated variant peptides of strongly immunodominant HLA-A2-restricted HIV gag epitope were able to bind HLA-A2, only one third were recognized by specific T cells (95). These two studies may give the rate of cross-reactivity for closely related peptides (21–33%) and could be particularly important in the context of a variable virus with an increased rate of mutations within epitopes (96).

Cross-reactive responses may be of clinical importance in the generation of pathology and autoimmunity. Several studies pointed that cross-reactivity may be the cause of increased immunopathology during successive unrelated viral infections (84–86) or as a result of application of T cell based therapy (97, 98). Expansion of cross-reactive T cell clones due to previous infections may underlie autoimmune diseases (99–101). Sometimes a pathogen epitope stimulates T cells in the context of a different MHC from the self-epitopes that react with these T cells, for example, Epstein-Barr virus EBN13-HLA-B8-specific cytotoxic T cells were shown to cross-react with a variety of self peptides presented by HLA-B35 (102). Together these observations point out that cross-reactive T cell responses might operate on different levels and much remains to be done to understand the extent of cross-reactivity and how it may differ in CD8, CD4, and regulatory populations of T cells.

## 4 Summary

We have reviewed current estimates of the T cell repertoire and identified their key limitations. Further progress will require the development of methods to determine the pairing of TCR *α* and *β* chains and thus more accurate quantification of the T cell diversity. Current estimates raise the puzzling question of why the potential repertoire is many orders of magnitude greater than the realized repertoire. We suggest that existing hypotheses are not able to explain this puzzle and have proposed an alternative hypothesis of “evolutionary sloppiness.”

One of the interesting observation that became obvious from our estimations is that precursor frequency per pathogen inherently links the TCR diversity and cross-reactivity which allows to predict the level of cross-reactivity between two random pathogens or unrelated peptides. Our estimates suggest that although cross-reactivity is a rare event for immunologically naïve individuals, probability to see the cross-reactive memory T cells becomes very high with an increase in successive infections.

Finally we note that we need to move from our current paradigm, which looks at each infection independent of other infections, to a more complex view that incorporates the terms for the interactions between the immune responses to different pathogens.

## Conflict of Interest Statement

The authors declare that the research was conducted in the absence of any commercial or financial relationships that could be construed as a potential conflict of interest.
